# Respiratory Co-Infections: Modulators of SARS-CoV-2 Patients’ Clinical Sub-Phenotype

**DOI:** 10.3389/fmicb.2021.653399

**Published:** 2021-05-28

**Authors:** Priyanka Mehta, Shweta Sahni, Samreen Siddiqui, Neha Mishra, Pooja Sharma, Sachin Sharma, Akansha Tyagi, Partha Chattopadhyay, A Vivekanand, Priti Devi, Azka Khan, Swati Waghdhare, Sandeep Budhiraja, Bharathram Uppili, Ranjeet Maurya, Vivek Nangia, Uzma Shamim, Pranjal P. Hazarika, Saruchi Wadhwa, Nishu Tyagi, Arun Dewan, Bansidhar Tarai, Poonam Das, Mohammed Faruq, Anurag Agrawal, Sujeet Jha, Rajesh Pandey

**Affiliations:** ^1^Genomics and Molecular Medicine Unit, CSIR-Institute of Genomics and Integrative Biology, New Delhi, India; ^2^Max Super Speciality Hospital (A Unit of Devki Devi Foundation), Max Healthcare, New Delhi, India; ^3^Academy of Scientific and Innovative Research, Ghaziabad, India

**Keywords:** co-infection, COVID-19, RVOP, holotranscriptome, metagenomics, respiratory viruses, anaerobic bacteria

## Abstract

Co-infection with ancillary pathogens is a significant modulator of morbidity and mortality in infectious diseases. There have been limited reports of co-infections accompanying SARS-CoV-2 infections, albeit lacking India specific study. The present study has made an effort toward elucidating the prevalence, diversity and characterization of co-infecting respiratory pathogens in the nasopharyngeal tract of SARS-CoV-2 positive patients. Two complementary metagenomics based sequencing approaches, Respiratory Virus Oligo Panel (RVOP) and Holo-seq, were utilized for unbiased detection of co-infecting viruses and bacteria. The limited SARS-CoV-2 clade diversity along with differential clinical phenotype seems to be partially explained by the observed spectrum of co-infections. We found a total of 43 bacteria and 29 viruses amongst the patients, with 18 viruses commonly captured by both the approaches. In addition to SARS-CoV-2, Human Mastadenovirus, known to cause respiratory distress, was present in a majority of the samples. We also found significant differences of bacterial reads based on clinical phenotype. Of all the bacterial species identified, ∼60% have been known to be involved in respiratory distress. Among the co-pathogens present in our sample cohort, anaerobic bacteria accounted for a preponderance of bacterial diversity with possible role in respiratory distress. *Clostridium botulinum*, *Bacillus cereus* and *Halomonas* sp. are anaerobes found abundantly across the samples. Our findings highlight the significance of metagenomics based diagnosis and detection of SARS-CoV-2 and other respiratory co-infections in the current pandemic to enable efficient treatment administration and better clinical management. To our knowledge this is the first study from India with a focus on the role of co-infections in SARS-CoV-2 clinical sub-phenotype.

## Introduction

The COVID-19 disease that emerged in Wuhan, China has spread across the globe in the past 1 year and assumed pandemic proportions. The infection is caused by Severe Acute Respiratory Syndrome Coronavirus-2 (SARS-CoV-2) which is a betacoronavirus of the Coronaviridae family (Zhu et al., 2020). Symptomatic patients present with a wide range of symptoms including fever, cough, runny nose, headache, nasal congestion and shortness of breath (Huang et al., 2020). While the majority of patients with SARS-CoV-2 infection have mild to moderate symptoms, some progress to a severe disease category despite standard treatment regime (World Health Organization, 2020). Patients with severe manifestation of the disease require Intensive Care Unit (ICU) admission, necessitated by the development of pulmonary pathology including ground glass opacification of the lungs and the Acute Respiratory Distress Syndrome (ARDS), often culminating in multiple organ failure and death (Fan et al., 2020; Wang et al., 2020).

Due to the diversity in clinical manifestations of the disease, more than one factor is assumed to possibly affect the clinical course of SARS-CoV-2 infection. Studies so far have indicated the role of older age, male gender and presence of comorbidities such as diabetes and hypertension as well as demographic and clinical factors in increasing the risk of developing a severe form of COVID-19 (Richardson et al., 2020; Wu and McGoogan, 2020; Zhou et al., 2020). Simultaneously, a limited number of reports have indicated the presence of co-infections with viral and bacterial respiratory pathogens in SARS-CoV-2 infected individuals. Studies have indicated the co-occurrence of respiratory viruses including Influenza virus, Human metapneumovirus, Rhinovirus and Respiratory syncytial virus in COVID-19 patients (Ding et al., 2020; Kim et al., 2020; Lin et al., 2020). Bacterial co-infections also affect the morbidity and mortality in viral respiratory infections. In this context, it is important to differentiate between hospital-acquired co-infection/secondary bacterial infection which develops during the course of hospitalization, versus an existing bacterial co-infection which is present when a patient reports to the hospital. A meta-review by Langford et al. studied the rate of bacterial co-infection in COVID-19 patients. Among the bacterial pathogens, the ones reported in SARS-CoV-2 positive patients include *Haemophilus influenzae*, *Pseudomonas aeruginosa*, *Klebsiella* spp. and *Mycoplasma* spp. (Langford et al., 2020). Most of these findings are, however, associative in nature and do not clearly indicate whether co-infection is a driver of poor clinical outcomes, or simply more common in severe categories of patients (e.g., intubation associated pneumonia in patients on ventilatory support).

To be able to better understand the COVID-19 pathogenesis and prognosis, it is important to elucidate the role of co-infections and concomitant interactions between co-infecting pathogens. Next generation sequencing (NGS) has aided the characterization and analysis of the genomic profile of not just the primary pathogen, i.e., SARS-CoV-2, but also of the associated microbiome, using the nucleic acids extracted from the respiratory specimens of patients (Chen et al., 2020; Peddu et al., 2020). The current magnitude of the problem requires multi-modal efficient management of the COVID-19 disease. Early detection of the co-infection with possible role in disease severity and outcome would be helpful in prioritising medical care. Identification of co-infections in SARS-CoV-2 positive individuals, especially of the bacterial species, may be crucial for better risk stratification of patients, disease prognosis and effective treatment, especially in context of the usage of suitable antibiotics.

## Materials and Methods

### Sample Collection and Processing

#### Sample Collection

The study was conducted by the CSIR-Institute of Genomics and Integrative Biology (CSIR-IGIB) in collaboration with the MAX Hospital, Delhi, India. Ethical clearance for the study was obtained from the Institutional Ethics Committee at the IGIB and the Max Hospital, respectively. A total of 100 patients with confirmed COVID-19 positive status, based on RT-PCR results, hospitalised in MAX Hospital were enrolled in the study. The nasopharyngeal and/or throat swabs along with a sputum sample were collected by the paramedical staff at the MAX Hospital on the day of reporting to the hospital. The tip of the swab was put into a vial containing 3 ml of Viral Transport Media (VTM) (HiViral Transport Kit, HiMedia, Cat. No: MS2760A-50NO), by breaking the applicator’s stick and sealing the tube tightly. The tube was then vortexed for 2 min to allow the dissolution of sample into the VTM solution followed by centrifugation and allowed to settle for some time before processing. For sputum samples, 200 μl of sputum was added to 200 μl of Sputum Liquefaction reagent, thoroughly mixed and incubated at 37°C for 10 min.

#### RNA Isolation and qRT-PCR Detection

Viral RNA from VTM solution or liquified sputum samples was extracted using commercially available RNA extraction kit (QIAmp viral mini kit, Qiagen, Cat. No. 52906). 200 μl of VTM solution or liquified sputum was processed for lysing and viral enrichment, in accordance with the kit protocol (QIAamp Viral RNA Mini Handbook). After washing with the wash buffers, viral RNA was eluted in RNase-free water. qRT-PCR for SARS-CoV-2 detection was performed using the TRUPCR SARS-CoV-2 kit (3B BlackBio Biotech India Ltd., Cat. No. 3B304). In brief, 10 μl of RNA was added to 15 μl of reaction mix in accordance with the kit protocol. The qRT-PCR was run on Rotor-Gene Q (Qiagen) using the recommended cycling conditions. The cycle threshold (Ct) value of 35 was considered for interpretation of the results.

### Sequencing

#### Respiratory Virus Oligo Panel (RVOP)

Double-stranded cDNA (ds cDNA) was synthesised from 100 ng of total RNA for all SARS-CoV-2 positive samples. The first strand of cDNA was synthesised using Superscript IV First strand synthesis system (Thermo Fisher Scientific, Cat. No. 18091050) followed by single-stranded RNA (ssRNA) digestion with RNase H for second strand synthesis using DNA Polymerase I Large (Klenow) Fragment (New England Biolabs, Cat. No. M0210S). The cDNA was purified using AMPure XP beads (AMPure XP, Beckman Coulter, Cat. No. A63881) and quantified using NanoDrop (ND-1000 UV-Vis Spectrophotometer, Thermo Fisher Scientific). 100 ng of purified ds cDNA was used for library prep using the Illumina DNA Prep with Enrichment kit (Illumina, Cat. No. 20018705). The process involves tagmentation followed by cleanup and amplification leading to indexed DNA fragments. Following tagmentation and indexing, enrichment was performed using the Illumina RVOP (Illumina, Cat no. 20042472), wherein 500 ng of each sample were pooled by mass in accordance with the reference guide (Illumina, Doc. No. 1000000048041v05) for the 12-plex hybridisation with biotinylated adjacent oligo-probes of the RVOP. The hybridisation was performed overnight after which the probes were captured by streptavidin-biotin based interactions. The final library was PCR amplified and purified before sequencing. The quality and quantity of the sequencing library was checked using Agilent 2100 Bioanalyzer with high sensitivity DNA chip and the Qubit dsDNA HS Assay kit, respectively. A loading concentration of 10 pM was prepared by denaturing and diluting the libraries in accordance with the MiSeq System Denature and Dilute Libraries Guide (Illumina, Document no. 15039740 v10). Sequencing was performed on the MiSeq system, using the MiSeq Reagent Kit v3 (150 cycles) at 2 × 75 bps read length.

#### Holo Transcriptome

Whole transcriptome sequencing was performed using the Illumina TrueSeq Stranded Total RNA Library Prep Gold (Illumina, Cat. No. 20020598). A total RNA input of 500 ng of each sample was depleted of cytoplasmic and mitochondrial rRNA by Ribo-Zero Gold, followed by fragmentation, first and second strand cDNA synthesis and purification with AMPure XP beads (AMPure XP, Beckman Coulter, Cat. No. A63881) in accordance with the reference guide (Illumina, Doc. No. 1000000040499 v00). The purified ds cDNA was adenylated at 3′ends, ligated with index adapters, which were further enriched by PCR based amplification. The final cDNA libraries were purified using AMPure XP beads and quantified using Qubit dsDNA HS Assay kit (Thermo Fisher Scientific, Cat. No. Q32854). The quality of cDNA libraries was checked by Agilent High Sensitivity DNA Kit using the Agilent 2100 Bioanalyzer. A final loading concentration of 400 pM was prepared by denaturing and diluting the libraries. Sequencing was performed on the NovaSeq 6000 system, using the NovaSeq SP reagents v1 at 2× 101 read length.

### Sequencing Data Analysis and Metagenomic Analysis

In this study, the total sample set was divided into two subsets with some overlapping samples across the two subsets. Fifty samples consisting of one subset were used for detection of viruses other than the SARS-CoV-2, using the Illumina Respiratory Virus Oligo Panel (RVOP). The second subset consisted of 48 clinical phenotype defined samples that were studied to explore the presence of microbes using Holo-Seq. Of the 48 samples, 21 samples were common with RVOP based study. The downstream analysis was automated using the Nextflow script. The methodology includes steps for base calling, quality check, removal of adapters (trimming), alignment, generation of consensus genome FASTA, and evaluation of species diversity, as well as coexistence and visualisation.

FastQC was used to check the Phred quality score for all sequences (Babraham Bioinformatics, 2020a – FastQC A Quality Control tool for High Throughput Sequence Data). For all samples, the quality score threshold was 20 and above. Adapter trimming was performed using the Trim Galore tool and alignment of sequences was performed using the HISAT2 algorithm on human data build hg38 (Kim et al., 2015; Babraham Bioinformatics, 2020b – Trim Galore!). To remove any human sequences from the dataset, SAMtools were used to remove aligned sequences (Li et al., 2009). Henceforth, only unaligned sequences were taken into consideration. BEDTools were used to generate consensus fasta and variant calling (Quinlan and Hall, 2010), which was followed by the alignment of sequences to the 30 respiratory virus panel of Illumina RVOP, to explore the presence of respiratory viruses other than the SARS-CoV-2. After alignment with the virus panel, the detected strains were counted using the number of reads mapped per strain. Kraken was used to assign taxonomic labels to microbial strains detected from the RVOP and the Holo-Seq analysis (Wood and Salzberg, 2014). The output from the metagenomic classification of the detected strains obtained from Kraken was analyzed further using the Pavian software (Breitwieser and Salzberg, 2020).

The Heatplus package from R was used to plot the heatmap (heatmaps, 2021: Flexible Heatmaps for Functional Genomics and Sequence Features version 1.14.0 from Bioconductor). For the alluvial plot, the Ggplot2 package from R was used and Plotly was used for the sunburst plot (Wickham, 2016 citation info) (Sievert, 2020). Cytoscape software was used to visualize the microbial presence captured by the RVOP and Holo-Seq analysis (Shannon et al., 2003).

### Statistical Analysis

All the data were summarized using descriptive statistics, wherein continuous variables are presented as median or interquartile range, and categorical variables are presented as percentages or proportions. A correlation analysis was conducted using the metadata of all 100 samples and plotted using the R corrplot package (GitHub, 2020 – taiyun/corrplot: Package corrplot is for visualizing a correlation matrix). We used the Mann–Whitney *U* test and Chi-square tests to compare the differences, wherever appropriate. The Kruskal Wallis test was used to compare the distribution of bacterial presence across our patient subgroups. The Shannon Diversity index (H) was calculated to characterise the bacterial species diversity as aerobes and anaerobes, in 48 patient samples used for the Holo-transcriptomics study, to account for the abundance and evenness of bacterial species in each patient sample (Supplementary Material).

### SARS-CoV-2 Phylogenetic Analysis

The phylogeny study was automated using NGphylogeny.fr online tool (Lemoine et al., 2019). Multiple sequence alignment was performed using the MAFFT algorithm for SARS-CoV-2 isolates from 100 samples using MN908947.3 as the reference SARS-CoV-2 sequence. The alignment was curated using trimAI. The phylogenetic tree was constructed using PhyML maximum likelihood algorithm as the statistical method. The phylogenetic analysis was visualized using FIGTREE software (FigTree, 2020). Gviz and trackViewer packages from R were used to plot the lollipop plot to visualise the mutations observed in 100 samples (Hahne and Ivanek, 2016; Ou and Zhu, 2019). Inkscape was used to modify the figures.

## Results

**FIGURE 1:**
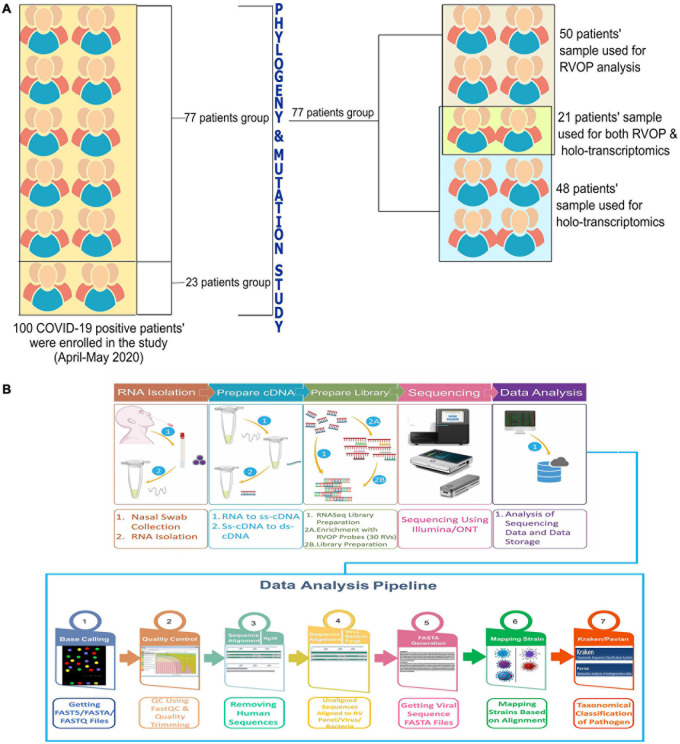
Schematic for experimental design of the study. **(A)** Patient sample distribution across clinical parameters of 100 COVID-19 individuals was analyzed for prioritizing samples for the Illumina RVOP (50 samples) and Holo-Seq (48 samples) with overlap of 21 samples. SARS-CoV-2 genome sequencing of all the 100 samples was performed. **(B)** Experimental workflow for sample collection, nucleic acid isolation, library preparation, sequencing, data analysis and identification of viral and bacterial species using a combination of sequencing strategies, i.e., RVOP and Holo-Seq.

The objective of the study was to explore and elucidate the correlation/association between differential presence of co-infecting microorganisms and, diversity in clinical symptoms as well as disease severity manifested by SARS-CoV-2 infected individuals. For identification of co-infecting species we used two different NGS approaches: target amplification based on hybridisation capture (Illumina RVOP) and whole transcriptome RNA-Seq (Holo-seq). RNA samples from 50 patients with COVID-19 were used for Illumina RVOP analysis followed by Holo-Seq of 48 samples with an overlap of a subset of 21 samples which were used for both the analyses (Figure 1). The selection of 77 unique patient samples from among the 100 enrolled in the study, for further analysis via RVOP and Holo-Seq, was based on the clinical sub-categorisation of the patient metadata.

### Demographics and Spectrum of Clinical Features in COVID-19 Patients

**FIGURE 2:**
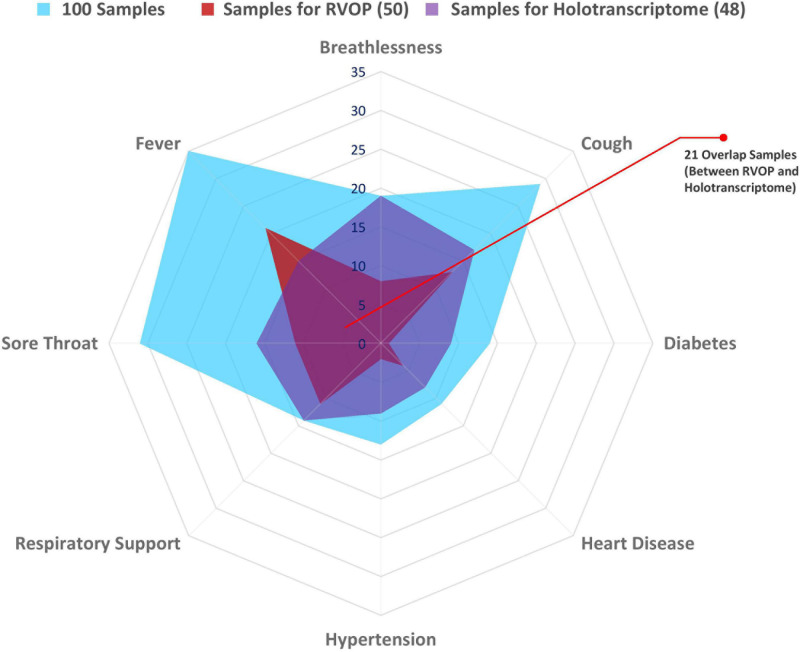
Spectrum of clinical symptoms for the COVID-19 patients in the study. Spider web plot representing the sample subsets used for RVOP (red) and Holo-Seq (purple) indicating sample overlap (magenta), along with distribution of clinical symptoms and comorbidities in all 100 patients (blue). Inter-individual variability in clinical symptoms is a hallmark of the COVID-19 disease, fever, sore throat and cough being the most common manifestations, along with differential distribution in pre-existing comorbidities.

The spider web plot represents the distribution of patient samples included in the RVOP (represented in red) and Holo-seq (represented in purple) analysis with respect to their associated clinical symptoms and comorbidities (Figure 2). The common samples among two datasets (represented in magenta) have dual objectives of validating the findings from two different sequencing approaches and enabling new discoveries. The outermost blue block represents the distribution of clinical metadata with respect to the 100 patients enrolled in our study. The plot highlights that a comparatively fewer number of patients investigated using RVOP (red block) had breathlessness and heart disease than the patients profiled using Holo-seq (purple block).

The requirement of respiratory support has been one of the important features for defining the COVID-19 disease severity (Nicholson et al., 2020; Ñamendys-Silva, 2020). Thus, the patient cohort was sub-categorised into two groups viz. patients requiring respiratory support and patients without respiratory support (Table 1). The median age of the subgroup of COVID-19 patients who required respiratory support was 58 years, and that without respiratory support was 29 years, while the median age for the patient cohort as a whole was 32 years. Higher age in our patient group was significantly associated with respiratory support requirements with a *p*-value < 0.05 (Table 1). Female and male patients’ ratio in our cohort was almost similar, with females accounting for slightly more than 50% of all the patients within each subgroup. Fever was the most common symptom reported by a majority of the patients (69%), followed by cough and sore throat that were reported by nearly one-third of the patients (33% each) (Figure 2 and Table 1). The loss of taste and smell was reported by five patients while only one of the patients was asymptomatic at admission (Table 1).

**TABLE 1:** Clinical characteristics of COVID-19 patients.

Characteristics	Overall (*n* = 90)*	No respiratory support (*n* = 76)	Respiratory support (*n* = 14)	
		Value	Value	*p*-value
**Age**	32(25–54)	30(24–50.5)	57.5(37.75–61.75)	**0.009^*a*^**
**Gender F/M**	44/46	37/39	7/7	0.042^*b*^
**Comorbidities**				
Heart disease	11(12.22)	8(10.52)	3(20)	0.048^*b*^
Diabetes	14(15.55)	11(14.47)	3(20)	0.066^*b*^
Hypertension	13(14.44)	13(17.10)	0	–
Thyroid	7(7.77)	6(7.89)	1(6.67)	0.655^*b*^
Asthma	2(2.22)	1(1.31)	1(6.67)	0.709^*b*^
Other comorbidities	14(15.55)	9(11.84)	5(33.33)	0.623^*b*^
**Clinical Parameters**				
RdRp	25.61(21.08–28.50)	25.51(21–28.4)	27.39(25.10–29.12)	0.070^*a*^
N	23(19–25.11)	22.38(19–25.11)	24.00(22.96–25.17)	0.397^*a*^
E	25(20.40–29)	24.42(20–28.71)	28.29(25.18–29.29)	0.196^*a*^
Temperature (°F)	98.2(97.7–98.6)	98.3(97.7–98.6)	98.2(98–98.8)	0.942^*a*^
SpO2	9.8(96–98)	98(96–98)	89(78–98)	**0.009^*a*^**
Pulse/min	86(78–98)	86(78–93)	85(78–100)	0.737^*a*^
**Symptoms**				
Sore throat	31(32.22)	28(36.84)	3(20)	0.418^*b*^
Breathlessness	19(21.11)	12(15.8)	7(46.67)	**0.003^*b*^**
Loss of taste and smell	5(5.55)	5(6.57)	0	–
Cough	29(32.22)	25(32.9)	4(26.67)	0.750^*b*^
Fever	62(68.9)	55(72.36)	7(46.67)	0.096^*b*^
Asymptomatic	1(1.11)	1(1.31)	0	–
Other Symptoms	33(36.67)	29(38.15)	4(26.67)	0.493^*b*^
**Hospital stay (in days)**	5(4–6)	5(4–5.25)	5.5(4–11.75)	0.385^*a*^

Data are shown as median (quartile) or n (%). ^*a*^Mann Whitney U test. ^*b*^χ^2^ test. *Data not available for all patients.

The peripheral oxygen saturation levels of patients at admission as measured by the SpO2 levels were found to be significantly higher in patients who did not require respiratory support than those who did require respiratory support (*p*-value = 0.009) (Table 1). Lower Oxygen saturation levels have been reported in more severe categories of COVID-19 patients (Chandra et al., 2020). We found that approximately 47% of the patients, who reported breathlessness at admission, went on to require respiratory support whereas only around 16% of those who did not report breathlessness, at admission, required respiratory support. The observed difference was statistically significant with a *p*-value < 0.05 (Table 1). While the median length of hospital stay was similar at 5 days across both the patient groups, the upper quartile range varied greatly, being much higher in the patient group who required respiratory support (Table 1).

**FIGURE 3:**
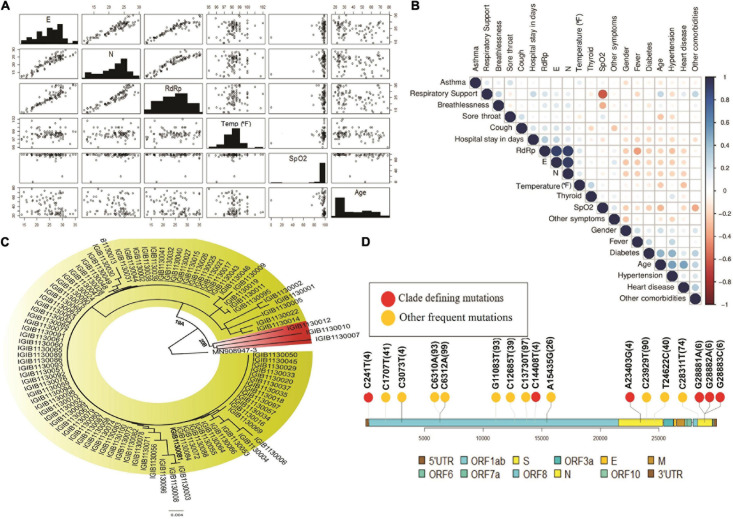
Diversity of patient features and SARS-CoV-2 clades. **(A)** Demographic and clinical characteristics of 100 COVID-19 patients highlighting the diversity of features inclusive of the age, gender, SpO2, fever and wide range of the Ct-values indicative of the viral load. **(B)** Correlation matrix for clinical and demographic features to capture possible association between the variables. **(C)** Phylogenetic classification of SARS-CoV-2 isolates with presence of two clades – 19A and 20B. **(D)** Lollipop plot with identified clade defining variants (red) as well as other high frequency variants (yellow).

To understand the correlation between different clinical parameters, we plotted the data using scatter plot and correlation matrix (Figures 3A,B, respectively). We found that patients in the older age group had lower Ct-values of SARS-CoV-2 RdRp and E gene, indicating a higher viral load when compared to younger patients (Figure 3A). Also, RdRp and E gene expression showed a negative correlation with fever, indicating that people with higher viral load had higher body temperature. We also observed that Ct values for RdRp, N, and E genes were negatively correlated with age, heart disease and other comorbidities (Figure 3B). Amongst all the parameters included in the study, presence of heart disease appears to be strongly correlated with higher SARS-CoV-2 viral load. From the correlation plot, we also observed that heart diseases were correlated with hypertension and other comorbidities, while hypertension and thyroid were closely associated with each other. We observed a strong negative correlation between the SpO_2_ levels and the requirement of respiratory support after hospitalisation.

### Limited Diversity of SARS-CoV-2 Clades

We performed the genome sequencing of SARS-CoV-2 isolates from all 100 patients to discover the viral clades as well as the phylogeny. We identified two clades according to the Nextclade classification, i.e., 19A and 20B (Figure 3C). Clade 19A, defined by positions 8782C (Nsp3) and 14408C (Nsp12/RdRp), was found in 97 out of the 100 patients. Clade 20B denoted by positions C3037T (Nsp3: 106F); A23403G (S: D614G); C14408T (Nsp12/RdRp: P4715L) and G28881A and G28882A (N: R203K) (Figure 3C) was found in only three of the 100 patients (represented in red). In addition to the clade defining variants, several other variants were identified within the viral isolates that occur at a high frequency (Figure 3D). These variants include nucleotide substitutions, some of which have been previously reported in the Indian cohort (preprint, Kumar et al., 2020; Sarkar et al., 2020) (Table 2).

**TABLE 2:** Frequency and description of the variants obtained from SARS-CoV-2 genome isolated from 100 patients.

Position/SNP	Gene	Amino acid change	Variant count
G11083T	Nsp6	L37F	93
C13730T	Nsp12/RdRp	A4489V	97
C6312A	Nsp3	T2016K	99
C6310A	Nsp3	S2015R	93
C23929T	S	T789T	90
C28311T	N	P13L	74
C1707T	Nsp2	S481F	41
G12685T	Nsp8	G4140H	39
T24622CA	S	–	40
A15435G	Nsp12/RdRp	–	26
TTTA21990T	S	–	20

The above observations seem to indicate that there is a limited viral genomic diversity in terms of the SARS-CoV-2 clades. However, a larger diversity of symptoms and severity in SARS-CoV-2 positive individuals suggests a possibility of a *missing link*, which might help fill in the gap. Given the fact, that the presence of co-infections during SARS-CoV-2 is still being understood with only a limited number of studies till date, we thought that it would be important to explore and elucidate the role and presence of co-infections in SARS-CoV-2 positive patients in the Indian context.

### Detection and Characterization of Respiratory Viruses Using RVOP

**FIGURE 4:**
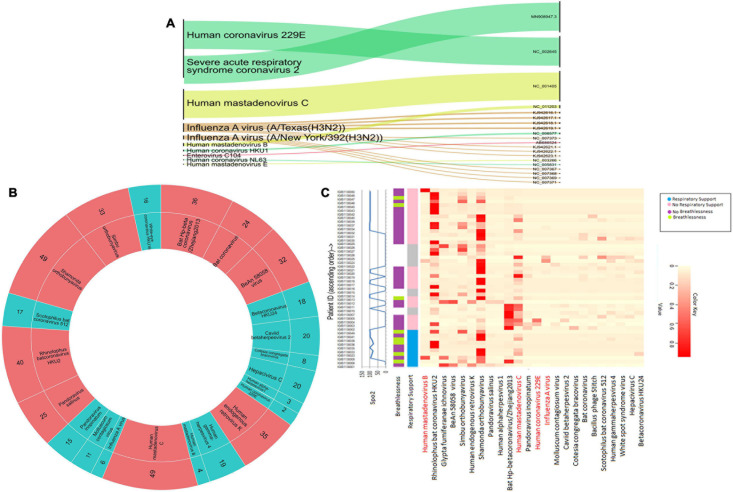
Viral diversity in nasopharyngeal tract of COVID-19 patients. **(A)** Using Illumina RVOP sequencing reads, predominant viral reads are mapped with reference genome. The relative thickness of the plot is indicative of the number of samples in which a particular virus is present. Thus, a thicker plot is indicative of presence in a higher number of samples. **(B)** Viral species identified in SARS-CoV-2 positive individuals along with the number of samples having a viral strain. Viruses found in ≤20 samples are indicated in blue and those in >20 samples are depicted in red. **(C)** Differential abundance of different viral species across individual patients along with their clinical manifestations including respiratory support, breathlessness and SpO2 levels.

We conducted a target enrichment based sequencing of RNA isolates from 50 SARS-CoV-2 positive individuals using Illumina RVOP panel. Alluvial plot depicts the reads that mapped to 10 predominant viral strains identified by the HISAT aligner (left panel) along with the NCBI ID for reference genome (right panel) (Figure 4A). Human Coronavirus 229E and Human mastadenovirus C had the most number of reads that mapped to RVOP viral strains, as depicted by the respective band-width. Two different strains of Influenza A virus viz. Texas and New York strains were identified in our samples.

Though we detected 10 different viral strains, the coverage of the aligned genome from the identified viruses was below 50%. To delve further, we used taxonomic sequence classifier, Kraken, to identify the overall diversity of co-infecting viral strains. Pavian was used to visualize the output from Kraken. For analysis, we included only those viruses whose relative abundance across at least one sample exceeded more than 1% of the total read count. Applying this cutoff value, we identified 26 viruses whose presence was detected across 50 samples (Figure 4B). Of all the viruses detected, Human mastadenovirus C and Shamonda orthobunyavirus were the most common viruses co-occurring with SARS-CoV-2, found in 49 of the 50 samples (Figure 4B).

Following the identification of viral species, we tried to correlate the differential presence of respiratory viruses in patients with their clinical parameters. A heatmap for differential presence of viruses across patients was plotted using the Heatplus package. We investigated whether levels of SpO2, condition of breathlessness and requirement of respiratory support across our samples are correlated with viral diversity (Figure 4C). It was observed that most of the patients who came to hospital with breathlessness required respiratory support later. Rhinophilus bat coronavirus HKU2 and Shamonda orthobunyavirus were present in the majority of patients with respiratory support. We found that Bat Hp-betacoronavirus was largely confined to patients who did not require respiratory support. Influenza A virus was seen in lower abundance in our patient group (Figure 4C).

### Differential Bacterial Abundance in COVID-19 Patients

**FIGURE 5:**
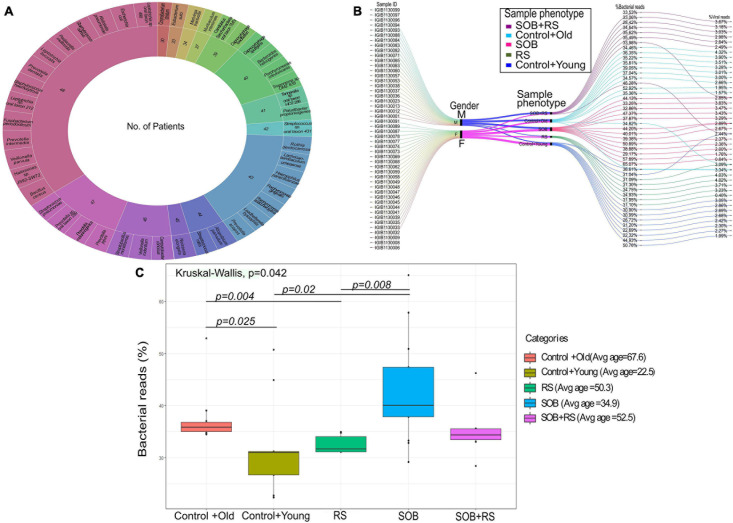
Bacterial abundance in the clinical symptoms sub-group of COVID-19 patients. **(A)** Bacterial species identified in COVID-19 patients along with the number of patients harbouring each species. The outer circle enumerates the bacterial species whereas the inner circle mentions the number of samples in which a particular species is present. **(B)** Cumulative viral and bacterial abundance (reads %age) in each patient across the five clinical subgroups designated based on respiratory symptoms and age. The gender, clinical sub-phenotype and the differential presence of the bacterial and the viral reads has been represented with different colour codes for each sub-group. **(C)** Kruskal–Wallis test for distribution of bacterial reads as a function of different clinical subgroups. The clinical sub-groups along with average age within the group has been plotted with significance calculation between the comparison sets.

To further our understanding of the possible modulators of COVID-19 disease severity, in addition to the respiratory viruses, we looked for the presence of other co-infecting bacteria in a set of 48 SARS-CoV-2 positive samples using Holo-seq. Kraken was used to identify the bacterial species within the sample set. Interestingly, the majority of bacterial species identified in our study were present in more than 40 patients, albeit with a differential abundance across patients. To understand the bacterial diversity within the nasopharyngeal environment of the SARS-CoV-2 patients, we plotted a sunburst plot of the identified bacterial species along with the frequency of samples harbouring those species (Figure 5A). It is important to note that many known pathogenic species such as *Clostridium botulinum*, *Bacillus cereus*, *Pseudomonas aeruginosa*, *Klebsiella pneumoniae* and *Neisseria gonorrhoeae* were discovered in almost all samples. We, thus, looked into sub-clinical classification of the SARS-CoV-2 positive individuals and checked if the differential abundance of bacteria would help explain the observed variation in clinical symptoms. The patients who had respiratory distress symptoms were divided into three subgroups based on the report of shortness of breath (SOB) at admission and/or requirement of respiratory support during the hospital stay (RS). The other group of patients who didn’t have any respiratory symptoms or requirements of respiratory support were classified into two control groups with differing age (Table 3).

**TABLE 3:** Sub-clinical classification of COVID-19 patient sub-groups.

Category	No. of samples	Avg. age	Shortness of breath (SOB)	Respiratory support (RS)
SOB+RS	8	52.5	Yes	Yes
RS	6	50.3	No	Yes
SOB	13	34.9	Yes	No
*Control+old	11	67.6	No	No
*Control+young	10	22.5	No	No

*The patients below the age of 50 who did not require respiratory support or showed signs of shortness of breath were grouped as control+young, whereas, patients above the age of 50 with similar clinical history were grouped as control+old.

For individual patients in all the five groups, we calculated the percentage of cumulative bacterial reads (%) as well as viral reads (%) (Figure 5B). The percentage of bacterial abundance varied from 26% to over 65% while the viral abundance varied from 0.4% to a little over 4%. Kruskal–Wallis test was used to determine the statistical significance of the distribution of bacterial presence across our patient subgroups (Figure 5C). Bacterial abundance was found to be significantly higher in the control+old group which had an average age of 67.5 years (37.47%) compared to the control+young group of patients with an average age of 22.5 years (32.8%) (*p*-value = 0.025). The bacterial presence in the control+old group (31%) differed significantly from that in the RS group (31.62%), though both lie within the higher age bracket (*p*-value = 0.004). We also found a significant difference in the bacterial abundance between control+young (35.81%) and SOB (40.01%) groups despite both falling in the lower age bracket (*p*-value = 0.02). Also, the bacterial presence within the RS and SOB groups differed significantly, with the latter showing a higher bacterial presence (*p*-value = 0.008). Interestingly, the average bacterial read count in the SOB group at 45.85% was much higher than the other four groups including the SOB+RS group, which had an average bacterial read count of 35.12%, although not statistically significant.

### Validation of Microbial Abundance/Diversity in a Subset of COVID-19 Patients

**FIGURE 6:**
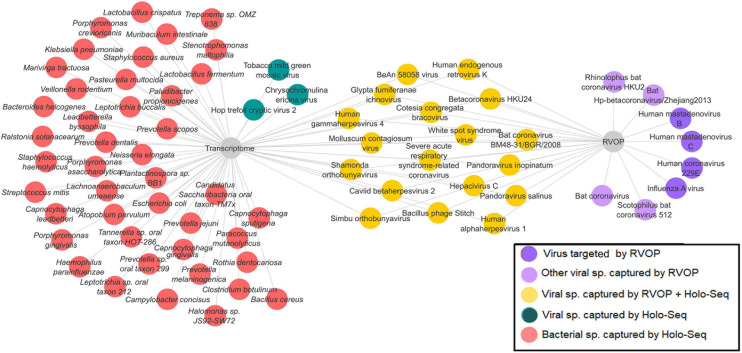
Overlap and unique viral and bacterial species in RVOP and Holo-Seq. Different colour codes have been used to highlight the unique bacterial and viral reads captured by the RVOP and the Holo-seq as well as the common findings between the two metagenomics methods. The unique and overlap set of bacteria and virus highlights the strength and limitations of each approach as well as complimentary strength of the experimental approach.

In a subset of 21 patients, microbial diversity was explored through both Holo-seq and RVOP, in correlation with clinical sub-phenotype diversity. We set up a cutoff value of >5% relative abundance for bacteria and >1% relative abundance of viral species for inclusion in our analysis. The threshold value was selected based on the required confidence for species identification and to exclude minimal sequence similarity with related organisms. In this subset, we identified 42 bacterial species (red nodes) and 21 viral species (blue and yellow nodes) using the Holo-seq (Figure 6). When we looked into the RVOP dataset 26 viral species were identified (Figure 4B). Interestingly, an overlap of 18 viral species was observed between both Holo-seq and RVOP (yellow nodes) (Figure 6). This highlights that inferences drawn using both the metagenomics approaches would provide complementary strength for studies of this dimension.

### Functional Classification of Co-infecting Viral and Bacterial Species

**FIGURE 7:**
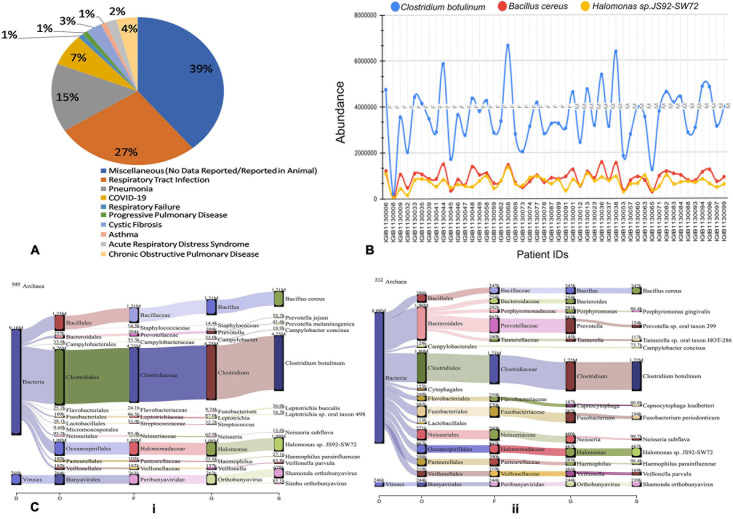
Possible functional role of co-infecting bacteria and viruses. **(A)** Percentage of identified bacterial species classified according to pathological roles Almost 60% of the identified bacterial species have literature pointing toward their role in respiratory distress ranging from respiratory tract infection to asthma and COPD. **(B)** Differential abundance of three anaerobic bacteria across patients based on read count. *Clostridium botulinum, Bacillus cereus* and *Halomonas* spp. are present in all the 48 Holo-seq samples but with differential read count. **(C)** Sankey plot representation of bacterial and viral taxonomic classification in (i) patient with respiratory support (ii) patient without respiratory support. Differential aerobe–anaerobe distribution may have a functional relevance in disease outcome in terms of respiratory distress. It is indicative of possible functional relevance in patient specific respiratory distress with an important role in disease outcome.

To further understand the role of viruses and bacteria in modulating the disease severity, we looked into the literature for the functional role of the viral and bacterial species that were identified in our study. Respiratory pathology being the hallmark of COVID-19 disease severity, we especially looked for their etiological relevance to respiratory infections and distress (Supplementary Table 1). Importantly, we observed that 27% of the viral species identified in our study have been previously associated with respiratory tract infection while 15% of them are causative agents for Pneumonia (Figure 7A). A small fraction of the identified viruses are also implicated in other pulmonary pathologies such as asthma, COPD, ARDS and Cystic Fibrosis. Majority of the identified species, however, had no known pathological relevance in humans but are known to be pathologically associated with other animals.

We visualized the composition of bacterial diversity in patients in terms of aerobic vs. anaerobic species (Supplementary Figure 1). The Shannon Diversity Index for bacterial communities at the species level was calculated across the data set from Holo-seq of 48 patients (Supplementary Table 2). It was found that the diversity of anaerobic bacteria was significantly much higher than that of the aerobes across all patients. Amongst the anaerobic bacteria known to be involved in hypoxia, *Clostridium botulinum*, *Bacillus cereus* and *Halomonas* spp. were found in all the samples (Figure 7B). Most of the peaks correspond to the patients who required respiratory support during their course of treatment (Patient Id: IGIB1130038, IGIB1130036, IGIB1130044). However, there were significant outliers in terms of those patients who did not require respiratory support despite showing very high presence of anaerobic bacteria (Patient Id: IGIB1130068 and IGIB1130048). At the other end of the spectrum were patients who have extremely low presence of anaerobes and yet required respiratory support (Patient Id: IGIB1130008). This finding would be especially relevant to explore in further studies, to understand the underlying cause for the observed disease severity in response to SARS-CoV-2 infection.

Finally, we also visualised the taxonomic distribution of identified bacterial and viral species in each individual patient through Sankey plots (Figure 7C). The left panel represents bacterial diversity in one of the patients who required respiratory support (Patient Id: IGIB1130006) [Figure 7C (i)]. It shows clear predominance of anaerobic bacterial species *Clostridium botulinum, Bacillus cereus* and *Halomonas* spp. The right panel is a Sankey plot visualisation of bacterial and viral species in a representative patient who did not require respiratory support (Patient Id: IGIB1130045) [Figure 7C (ii)]. These two plots are indicative of the spectrum of bacterial diversity with possible role in disease severity (respiratory distress). However, this does not suggest any conclusive representation with respect to the association of any particular pathogen with either group of patients. An analysis of all the individual patients did not reveal any distinct pattern in distribution of bacterial and viral species among the two groups of patients.

## Discussion

**FIGURE 8:**
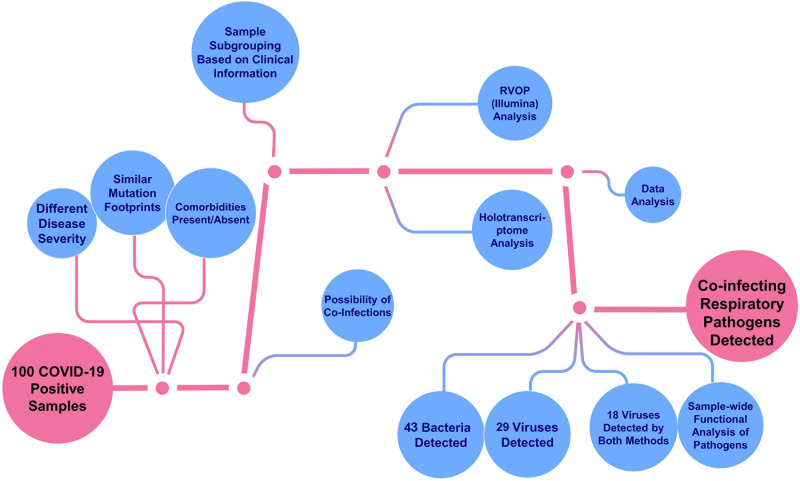
Metagenomics based identification of co-infecting pathogens in COVID-19 patients. It summarizes the whole study, highlighting the variables in deducing the functional inferences from the data. With multiple variables at every stage, from sample collection to sequencing to functional interpretation, it highlights the importance of an integrative approach towards understanding the host–pathogen interaction outcome in the course of an infection.

The present study utilized two different metagenomic approaches to identify the diversity of co-infecting species present within the upper respiratory tract of COVID-19 patients from the Delhi-NCR (National Capital Region) during the initial phase of the epidemic in India (April–May 2020). Metagenomic sequencing offers advantages over other targeted methods of pathogen detection by factoring out the limitations associated with ascribing a particular disease state to a single causative agent. It allows autarkic verification of the primary pathogen along with the underlying microbiome which may be playing an important role in the disease progression and prognosis. Out of the three potential core elements that determine the course and end-point of an infection, our study tried to capture the possible role of two, i.e., pathogenic variation and the airway microbiome of the host (the third one being the host transcriptional response). To our knowledge, this is the first study from India aiming to elucidate the role of co-inhabiting species (virus and bacteria) in modulating the severity and trajectory of SARS-CoV-2 infection (Figure 8).

To appreciate the role of nasopharyngeal microbiome in affecting the SARS-CoV-2 disease course, we compared the demographic and associated clinical parameters in two sub-groups of patients who required or who did not require respiratory support during their stay in the hospital. The dichotomy in the requirement of respiratory support showed a correlation with older age. This is in sync with the previous studies which have reported older age as a risk factor for COVID-19 severity owing to weakened immune responses (Wu C. et al., 2020). The presence of breathlessness and lower SpO2 levels at hospital admission also correlated with the requirement of respiratory support in our patient group. SpO2 level lower than 90% is a well-known indicator of hypoxaemia and respiratory distress (Majumdar et al., 2011). Chandra et al. reported a case of “Silent Hypoxia”, which involves severe hypoxaemia without proportional signs of respiratory distress. Such patients rapidly progress to respiratory decompensation, underscoring the need to stringently monitor SpO2 levels as part of community surveillance, to identify apparently clinically healthy patient suspects of COVID-19 (Chandra et al., 2020).

Within our samples, we identified two clades of SARS-CoV-2 virus, viz. 19A and 20B. 19A is the ancestral haplotype with presumed origin in China. A majority of the viral isolates from our cohort belonged to clade 19A, which has been previously reported to be mainly present in Northern India. Clade 20B with origin from European nations, along with other A2a haplotypes, became dominant in India after June 2020 (preprint, Maitra et al., 2020). Although this was found to be less frequent in our sample set, a possible reason may be the time of sample collection which predated June 2020. D614G (A23403G) mutation within Spike protein (S) has been associated with viral isolates of European descent and is found to co-occur with three other mutations at positions C241T, C3037T and C14408T, all of which, together form a haplotype (Korber et al., 2020). Though D614G was not very frequent in our sample set, it has been reported to be associated with enhanced infectivity, competitive fitness and transmission (Hou et al., 2020; Plante et al., 2020). Another missense SNP at position C6312A within Nsp 3 protein was observed in 99% of the viral isolates in our sample set. This mutation is found to be predominant in viral isolates from India and is a defining variant for clade I/A3i (Raghav et al., 2020).

Concurrent infections are known to modulate the severity and outcome of a disease including comorbidity and mortality. Many recent papers have highlighted the co-occurrence of respiratory pathogens including bacteria and viruses in SARS-CoV-2 infected individuals (Massey et al., 2020). An investigation into the role of co-infecting pathogens within the respiratory tract was primarily guided by the observation that viral genomics and patient demographics cannot adequately explain the variability seen in the COVID-19 disease course in terms of the requirement of respiratory support and disease outcome. Respiratory co-infection biology also assumes importance in the context of the seasonal change in India, wherein winter weather is typically associated with respiratory illnesses caused by viruses and bacteria other than SARS-CoV-2 which present similar disease manifestations (Fares, 2013).

We used nasopharyngeal swab as a proxy for respiratory tract microbiota for identifying co-pathogens within COVID-19 patients. Unlike many other studies reporting the co-occurrence of Influenza virus with SARS-CoV-2, we did not find any significant presence of Influenza virus infection in our samples (Ding et al., 2020; Ozaras et al., 2020; Wu X. et al., 2020). Influenza is known to manifest a discrete seasonality which is also affected by the latitude of the region (Yaari et al., 2013; Koul et al., 2014). April-May in the Delhi-NCR is not a time for Influenza peak; this may possibly explain our observation with respect to the absence of Influenza co-infection in our samples. Among other respiratory viruses, Human mastadenovirus was present in most of our samples. Human mastadenoviruses have been reported to cause respiratory tract diseases, especially in children (Chen et al., 2015; Scott et al., 2016; Yao et al., 2019). Human Coronavirus 229E, found in some of our samples is known to be an opportunistic pathogen, causing life-threatening infections of the lower respiratory tract in immunocompromised individuals (Vassilara et al., 2018). Human gammaherpesvirus, which has been implicated in pulmonary fibrosis, was identified in over one third of the samples (Williams, 2014) (Supplementary Table 1).

**FIGURE 9:**
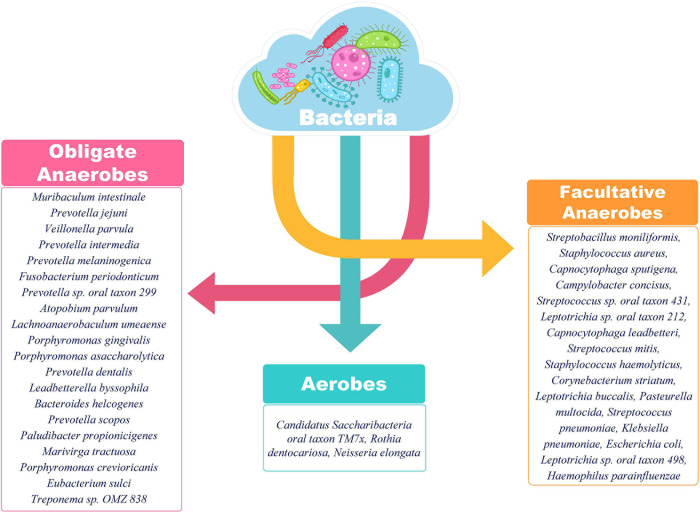
Classification of identified bacterial species according to their aerobic/anaerobic requirements. The preponderance of obligate and facultative anaerobes over aerobes is an important insight. The differential abundance is also an important aspect to be considered for functional interpretation in conjunction with the clinical sub-phenotype. A higher abundance of anaerobic bacteria is important to highlight the differential disease severity and respiratory distress due to SARS-CoV-2 infection.

Most of the bacterial species identified in SARS-CoV-2 positive individuals in our study have known roles in respiratory pathology (Supplementary Table 1). *Klebsiella pneumonia* acts as an opportunistic pathogen and affects the critically ill and immunocompromised individuals mainly. Besides pneumonia, it is also known to cause other health care related complications including urinary tract infections (UTIs) and bloodstream infections (Martin and Bachman, 2018). *Streptococcus pneumoniae* has long been known to cause community acquired pneumonia in populations of all age groups, elderly people are, however, more susceptible. It is known to show the worst prognosis in patients with a history of smoking or presence of comorbidities as asthma or COPD, same as is the case with SARS-CoV-2 (Pal et al., 2020). Interestingly, the majority of identified bacterial species in the respiratory microbiome of SARS-CoV-2 positive patients belonged to the category of anaerobic bacteria lending credibility to the hypothesis of *Happy Hypoxia* in COVID-19 (Figure 9) (Dhont et al., 2020; González-Duarte and Norcliffe-Kaufmann, 2020). The increased abundance of anaerobic bacterial species, especially *Prevotella*, has been proposed to cause degradation of haemoglobin, thereby, further affecting the course of hypoxia (preprints, Chakraborty, 2020; Chakraborty and Das, 2020). Anaerobic bacteria, particularly *Fusobacterium periodonticum*, is also known to be the cause of Ventilator associated Pneumonia (VAP) in mechanically ventilated patients (Robert et al., 2003). Altered balance of aerobes-anaerobes in COVID-19 patients has important implications for altering the treatment regimen to include targeted use of anaerobe-specific antibiotics.

We observed that different groups of patients that required respiratory support or did not require the same had differential abundance of bacteria. However, we will be cautious while extrapolating our observation with respect to bacterial abundance, to the observed differences in disease trajectory of individual patients. This is particularly important as the identified respiratory viruses and bacteria were part of the upper respiratory tract (URT) where most pathogenic species survive as commensals and become virulent only when they invade the lower respiratory tract.

In view of the magnitude of the problem and excessive burden on the healthcare workers, there are certain limitations with respect to sampling that limited the scope of the present study. First, the nasopharyngeal samples were obtained from the patients only at the time of admission and there was no longitudinal follow-up, which could have allowed a better evaluation of the dynamic change in nasopharyngeal microbiome during the course of SARS-CoV-2 infection, including, of hospital-acquired infections. Second, all the samples were restricted to Delhi-NCR region and, as there was a nationwide lockdown imposed in the country at the time, hence much variation in terms of viral genome and phylogeny cannot be expected in viral isolates. Finally, the dataset lacked any mortality cases.

The findings presented herewith have tried to correlate the role played by co-infecting respiratory pathogens in modulating the SARS-CoV-2 disease trajectory. The findings do not suggest a pan-study causal role for a particular co-infecting pathogen, but it does indicate possibilities of disease modulation in patient sub-groups. The findings also assume significance in the context of widespread usage of antibiotics during the current pandemic which may have long term impact in terms of increased antimicrobial resistance and emergence of multi-drug resistant strains. A further investigation into the role of co-infections in SARS-CoV-2 positive individuals in an extended dataset with matched SARS-CoV-2 negative controls is warranted to enable better understanding and management of the current COVID-19 disease pandemic.

## Data Availability Statement

The datasets presented in this study can be found online at the NCBI-SRA under the accession numbers PRJNA676016 and PRJNA678831, the consensus fasta are available at the GISAID-EpiCoV (https://www.gisaid.org/) under the submission IDs: EPI_ISL_459911-EPI_ISL_459944 and EPI_ISL_641320-EPI_ISL_641376, and the classification reports of RVOP and Holo-transcriptomics for all patient samples are available for visualization at https://doi.org/10.6084/m9.figshare.13560506.

## Ethics Statement

The studies involving human participants were reviewed and approved by CSIR-IGIB’s Human Ethics Committee Clearance (Ref No: CSIR-IGIB/IHEC/2020-21/01). The patients/participants provided their written informed consent to participate in this study.

## Author Contributions

PM, SSH, and SSA performed analysis, made figures, and wrote the manuscript. NM performed analysis and made figures. VA and BU performed analysis. PC made figures. SSI, AT, SWW, SB, VN, PPH, AD, BT, POD, and SJ contributed to the clinicians who diagnosed patients and shared samples with IGIB. PrD and AK contributed to the manuscript related literature survey. RM contributed to the data upload and helped in analysis. PS, US, SAW, NT, and MF performed genomic experiments. AA initiated collaboration and facilitated work. RP designed, conceptualized, implemented and coordinated the study, along with inferences of the results and wrote the manuscript. All authors contributed to the article and approved the submitted version.

## Conflict of Interest

The authors declare that the research was conducted in the absence of any commercial or financial relationships that could be construed as a potential conflict of interest.

## Supplementary Material

Supplementary Figure 1 Shannon Index plot showing bacterial abundance and evenness across patient samples.

Supplementary Table 1 Co-infecting pathogens and their role in respiratory tract infections.
