# Supplementary material for: Respiratory Co-Infections: Modulators of SARS-CoV-2 Patients’ Clinical Sub-Phenotype
Source: Front Microbiol. 2021 May 28;12:653399. doi: 10.3389/fmicb.2021.653399 (PMC8193731; doi:10.3389/fmicb.2021.653399)
Supplement: Supplementary Figure 1 — Shannon Index plot showing bacterial abundance and evenness across patient samples. [file Data_Sheet_1.zip › Supplementary Table 1.pdf]

Supplementary Table 1: Co-infecting pathogens and their role in respiratory tract infections

**Bacterial Species**	**Role in Respiratory tract infection**
Veillonella parvula	Lung abscess(Shah et al. 2008)
Veillonella rodentium	Respiratory tract infection in elderly patients(Saladi, Zeana, and Singh 2017)
Prevotella intermedia	Chronic inflammation(Nagaoka et al. 2014)
Prevotella melaninogenica	Resident microbe, aspiration pneumonia(Nagaoka et al. 2014)
Prevotella sp. oral taxon 299	Abundant in asthma control patients(Mathieu et al. 2018)
Rothia dentocariosa	Opportunistic pulmonary infection(A., M.Z., and B. 2017; HD and Thavarajah 2018)
Capnocytophaga sputigena	Resident oral microbe; opportunistic pathogen (Patel and Sampson 2020; Gosse et al. 2019)
Capnocytophaga leadbetteri	Severe community-acquired pneumonia in HIV patient(Fossé et al. 2018)
Campylobacter concisus	Resident oral microbe;<br>pulmonary abscess in immune-compromised patients(Hagemann et al. 2018)
Fusobacterium periodonticum	Ventilator associated pneumonia(de Carvalho Baptista et al. 2018)
Atopobium parvulum	Involved in Cystic Fibrosis Pulmonary Disease(Badri et al.2019)
Haemophilus parainfluenzae	Upper respiratory tract contaminant, cause severe COPD(Nik Zuraina et al. 2018)
Porphyromonas gingivalis	Causes inflammation in presence of H1N1(X. Li et al. 2018; Chen et al. 2018)
Streptococcus mitis	Acute respiratory distress syndrome in cancer patients(Bochud, Calandra, and Francioli 1994)
Streptococcus pneumoniae	Community acquired pneumonia (Bennett 1994)
Staphylococcus haemolyticus	Reported co-pathogen with SARS-CoV-2 in patients with co-morbidities(Lv et al. 2020)
Staphylococcus aureus	Pulmonary infection(Parker and Prince 2012)
Corynebacterium striatum	Causes chronic obstructive pulmonary disease(F. et al. 2014; Keske et al. 2020)
Leptotrichia buccalis	Opportunistic pathogen("Serious Infection with Leptotrichia Buccalis. Report of a Case and Review of the Literature" 1980)
Leptotrichia sp. oral taxon 498	serious pneumonia in immune-compromised and immune-competent patients(Kawanami et al. 2009)
Pasteurella multocida	Non-specific clinical course; co-pathogen in respiratory tract infection(N. and H. 2018)
Klebsiella pneumonia	Opportunistic pathogen in immune-compromised patients(Martin and Bachman 2018; Nik Zuraina et al. 2018; Zhu et al. 2020)

Escherichia coli	Hospital acquired pneumonia(De Lastours et al. 2015)
**Viral strains**	
Human mastadenovirus B<br>Human mastadenovirus C	Associated with respiratory tract infections(Girardi et al. 2019b; 2019a)
Human alphaherpesvirus 1<br>Human gammaherpesvirus 4	Causative agent of oral herpes, pneumonia disease.(Van Cleemput et al. 2020)<br>plays a role in development of lung fibrosis(Williams 2014)
Human coronavirus 229E	Immune-compromised patients have been reported with severe and life-threatening LRTIs(Vassilara et al. 2018)
Influenza A virus	Mostly mild infections, however some present as<br>severe acute respiratory infection(X. Li et al. 2018; Zhu et al. 2020; Pariani et al. 2014)
Bat coronavirus	Bat borne corona viruses have caused several emerging infectious disease outbreaks of global significance, including SARS.(H. Li et al. 2019)

A., El-bakush, Rizwan M.Z., and Kabchi B. 2017. "Rothia Mucilaginosa Pneumonia in an Immunocompetent Patient Diagnosed by Bronchoscopy and Fine Needle Aspiration." *American Journal of Respiratory and Critical Care Medicine*.

Badri, M., Nilson, B., Ragnarsson, S., Senneby, E., and Rasmussen, M. (2019). Clinical and microbiological features of bacteraemia with Gram-positive anaerobic cocci: a population-based retrospective study. Clin. Microbiol. Infect. 25, 760.e1–760.e6. doi: 10.1016/j.cmi.2018.09.001

Bennett, Barbara B. 1994. "Community-Acquired Pneumonia." *Primary Care Update for Ob/Gyns*. https://doi.org/10.7326/0003-4819-151-7-200910060-01004.

Bochud, Pierre Yves, Thierry Calandra, and Patrick Francioli. 1994. "Bacteremia Due to Viridans Streptococci in Neutropenic Patients: A Review." *The American Journal of Medicine*. https://doi.org/10.1016/0002-9343(94)90009-4.

Carvalho Baptista, Ivany Machado de, Frederico Canato Martinho, Gustavo Giacomelli Nascimento, Carlos Eduardo da Rocha Santos, Renata Falchete do Prado, and Marcia Carneiro Valera. 2018. "Colonization of Oropharynx and Lower Respiratory Tract in Critical Patients: Risk of Ventilator-Associated Pneumonia." *Archives of Oral Biology*. https://doi.org/10.1016/j.archoralbio.2017.09.029.

Chen, Yongju, Rui Zhou, Zhe Yi, Yonggang Li, Ying Fu, Yibo Zhang, Ping Li, Xin Li, and Yaping Pan. 2018. "Porphyromonas Gingivalis Induced Inflammatory Responses and Promoted Apoptosis in Lung Epithelial Cells Infected with H1N1 via the Bcl-2/Bax/Caspase-3 Signaling Pathway." *Molecular Medicine Reports*. https://doi.org/10.3892/mmr.2018.8983.

Cleemput, Jolien Van, Katrien C. K. Poelaert, Kathlyn Laval, Nathalie Vanderheijden, Maarten

Dhaenens, Simon Daled, Filip Boyen, Frank Pasmans, and Hans J. Nauwynck. 2020. "An Alphaherpesvirus Exploits Antimicrobial β-Defensins To Initiate Respiratory Tract Infection." *Journal of Virology*. https://doi.org/10.1128/jvi.01676-19.

F., Renom, Gomila M., Garau M., Gallegos M.D.C., Guerrero D., Lalucat J., and Soriano J.B. 2014. "Respiratory Infection by Corynebacterium Striatum: Epidemiological and Clinical Determinants." *New Microbes and New Infections*.

Fossé, Q., C. Flateau, C. Gomart, J. W. Decousser, and S. Gallien. 2018. "Severe Community-Acquired Capnocytophaga Leadbetteri Pneumonia in a HIV-Infected Patient." *Medecine et Maladies Infectieuses*. https://doi.org/10.1016/j.medmal.2017.11.003.

Girardi, Viviane, Tatiana Schäffer Gregianini, Juliana Schons Gularte, Meriane Demoliner, Claudete Farina Seadi, Selir Maria Straliotto, Letícia Garay Martins, and Fernando Rosado Spilki. 2019a. "Correction to: Temporal Dynamics of Human Mastadenovirus Species in Cases of Respiratory Illness in Southern Brazil (Brazilian Journal of Microbiology, (2019), 50, 3, (677-684), 10.1007/S42770-019-00084-4)." *Brazilian Journal of Microbiology*. https://doi.org/10.1007/s42770-019-00115-0.

———. 2019b. "Temporal Dynamics of Human Mastadenovirus Species in Cases of Respiratory Illness in Southern Brazil." *Brazilian Journal of Microbiology*. https://doi.org/10.1007/s42770-019-00084-4.

Gosse, Laurie, Sophie Amrane, Morgane Mailhe, Grégory Dubourg, and Jean Christophe Lagier. 2019. "Capnocytophaga Sputigena: An Unusual Cause of Community-Acquired Pneumonia." *IDCases*. https://doi.org/10.1016/j.idcr.2019.e00572.

Hagemann, Jürgen Benjamin, Stephanie Haverkamp, Beate Grüner, Florian Kuchenbauer, and Andreas Essig. 2018. "Pulmonary Campylobacter Concisus Infection in an Immunocompromised Patient with Underlying Mucormycosis." *International Journal of Infectious Diseases*. https://doi.org/10.1016/j.ijid.2018.08.021.

HD, Bachert, and K Thavarajah. 2018. "Rothia Mucilaginosa Pneumonia Complicating Interstitial Lung Disease." *American Journal of Respiratory and Critical Care Medicine*.

Kawanami, Toshinori, Kazumasa Fukuda, Kazuhiro Yatera, Takashi Kido, Chiharu Yoshii, Hatsumi Taniguchi, and Masamitsu Kido. 2009. "Severe Pneumonia with Leptotrichia Sp. Detected Predominantly in Bronchoalveolar Lavage Fluid by Use of 16S RRNA Gene Sequencing Analysis." *Journal of Clinical Microbiology*. https://doi.org/10.1128/JCM.01429-08.

Keske, Şiran, Süda Tekin, Bilgin Sait, Pelin İrkören, Mahir Kapmaz, Cansu Çimen, Semra Uğur, et al. 2020. "Appropriate Use of Tocilizumab in COVID-19 Infection." *International Journal of Infectious Diseases*. https://doi.org/10.1016/j.ijid.2020.07.036.

Lastours, Victoire De, Ryan E. Malosh, Allison E. Aiello, and Betsy Foxman. 2015. "Prevalence of Escherichia Coli Carriage in the Oropharynx of Ambulatory Children and Adults with and without Upper Respiratory Symptoms." *Annals of the American Thoracic Society*. https://doi.org/10.1513/AnnalsATS.201412-586LE.

Li, Hongying, Emma Mendelsohn, Chen Zong, Wei Zhang, Emily Hagan, Ning Wang, Shiyue Li, et al. 2019. “Human-Animal Interactions and Bat Coronavirus Spillover Potential among Rural Residents in Southern China.” *Biosafety and Health*. https://doi.org/10.1016/j.bsheal.2019.10.004.

Li, Xin, Chen Li, Jun chao Liu, Ya ping Pan, and Yong gang Li. 2018. “In Vitro Effect of Porphyromonas Gingivalis Combined with Influenza A Virus on Respiratory Epithelial Cells.” *Archives of Oral Biology*. https://doi.org/10.1016/j.archoralbio.2018.04.003.

Lv, Zhihua, Shaohua Cheng, Juan Le, Jingtao Huang, Lina Feng, Binghong Zhang, and Yan Li. 2020. “Clinical Characteristics and Co-Infections of 354 Hospitalized Patients with COVID-19 in Wuhan, China: A Retrospective Cohort Study.” *Microbes and Infection*. https://doi.org/10.1016/j.micinf.2020.05.007.

Martin, Rebekah M., and Michael A. Bachman. 2018. “Colonization, Infection, and the Accessory Genome of Klebsiella Pneumoniae.” *Frontiers in Cellular and Infection Microbiology*. https://doi.org/10.3389/fcimb.2018.00004.

Mathieu, Elliot, Unai Escribano-Vazquez, Delphyne Descamps, Claire Cherbuy, Philippe Langella, Sabine Riffault, Aude Remot, and Muriel Thomas. 2018. “Paradigms of Lung Microbiota Functions in Health and Disease, Particularly, in Asthma.” *Frontiers in Physiology*. https://doi.org/10.3389/fphys.2018.01168.

N., Itoh, and Kurai H. 2018. “A Case of Pasteurella Multocida Pneumonia Needed to Differentiate from Non-Tuberculous Mycobacteriosis.” *IDCases*.

Nagaoka, Kentaro, Katsunori Yanagihara, Yoshitomo Morinaga, Shigeki Nakamura, Tatsuhiko Harada, Hiroo Hasegawa, Koichi Izumikawa, et al. 2014. “Prevotella Intermedia Induces Severe Bacteremic Pneumococcal Pneumonia in Mice with Upregulated Platelet-Activating Factor Receptor Expression.” *Infection and Immunity*. https://doi.org/10.1128/IAI.00943-13.

Nik Zuraina, Nik M.N., Abdullah Sarimah, Mohamad Suharni, Habsah Hasan, and Siti Suraiya. 2018. “High Frequency of Haemophilus Influenzae Associated with Respiratory Tract Infections among Malaysian Hajj Pilgrims.” *Journal of Infection and Public Health*. https://doi.org/10.1016/j.jiph.2018.07.010.

Pariani, Elena, Marianna Martinelli, Marta Canuti, Seyed Mohammad Jazaeri Farsani, Bas B. Oude Munnink, Martin Deijs, Elisabetta Tanzi, Alessandro Zanetti, Lia Van Der Hoek, and Antonella Amendola. 2014. “Influenza and Other Respiratory Viruses Involved in Severe Acute Respiratory Disease in Northern Italy during the Pandemic and Postpandemic Period (2009-2011).” *BioMed Research International*. https://doi.org/10.1155/2014/241298.

Parker, Dane, and Alice Prince. 2012. “Immunopathogenesis of Staphylococcus Aureus Pulmonary Infection.” *Seminars in Immunopathology*. https://doi.org/10.1007/s00281-011-0291-7.

Patel, Jay, and Victoria Sampson. 2020. “The Role of Oral Bacteria in COVID-19.” *The Lancet Microbe*. https://doi.org/10.1016/s2666-5247(20)30057-4.

Saladi, Lakshmi, Cosmina Zeana, and Manisha Singh. 2017. “ Native Valve Endocarditis Due to Veillonella Species: A Case Report and Review of the Literature .” *Case Reports in Infectious Diseases*. https://doi.org/10.1155/2017/4896186.

“Serious Infection with Leptotrichia Buccalis. Report of a Case and Review of the Literature.” 1980. *The American Journal of Medicine*. https://doi.org/10.1016/0002-9343(80)90440-4.

Shah, Ashok, Chandramani Panjabi, Vidya Nair, Rama Chaudhry, and S. S. Thukral. 2008. “Veillonella as a Cause of Chronic Anaerobic Pneumonitis.” *International Journal of Infectious Diseases*. https://doi.org/10.1016/j.ijid.2008.03.018.

Vassilara, Foula, Aikaterini Spyridaki, George Pothitos, Athanassia Deliveliotou, and Antonios Papadopoulos. 2018. “A Rare Case of Human Coronavirus 229E Associated with Acute Respiratory Distress Syndrome in a Healthy Adult.” *Case Reports in Infectious Diseases*. https://doi.org/10.1155/2018/6796839.

Williams, K. J. 2014. “Gammaherpesviruses and Pulmonary Fibrosis: Evidence From Humans, Horses, and Rodents.” *Veterinary Pathology*. https://doi.org/10.1177/0300985814521838.

Zhu, Xiaojuan, Yiyue Ge, Tao Wu, Kangchen Zhao, Yin Chen, Bin Wu, Fengcai Zhu, Baoli Zhu, and Lunbiao Cui. 2020. “Co-Infection with Respiratory Pathogens among COVID-2019 Cases.” *Virus Research*. https://doi.org/10.1016/j.virusres.2020.198005.
