# Supplementary figures and images for: Respiratory Co-Infections: Modulators of SARS-CoV-2 Patients’ Clinical Sub-Phenotype
Source: Front Microbiol. 2021 May 28;12:653399. doi: 10.3389/fmicb.2021.653399 (PMC8193731; doi:10.3389/fmicb.2021.653399)

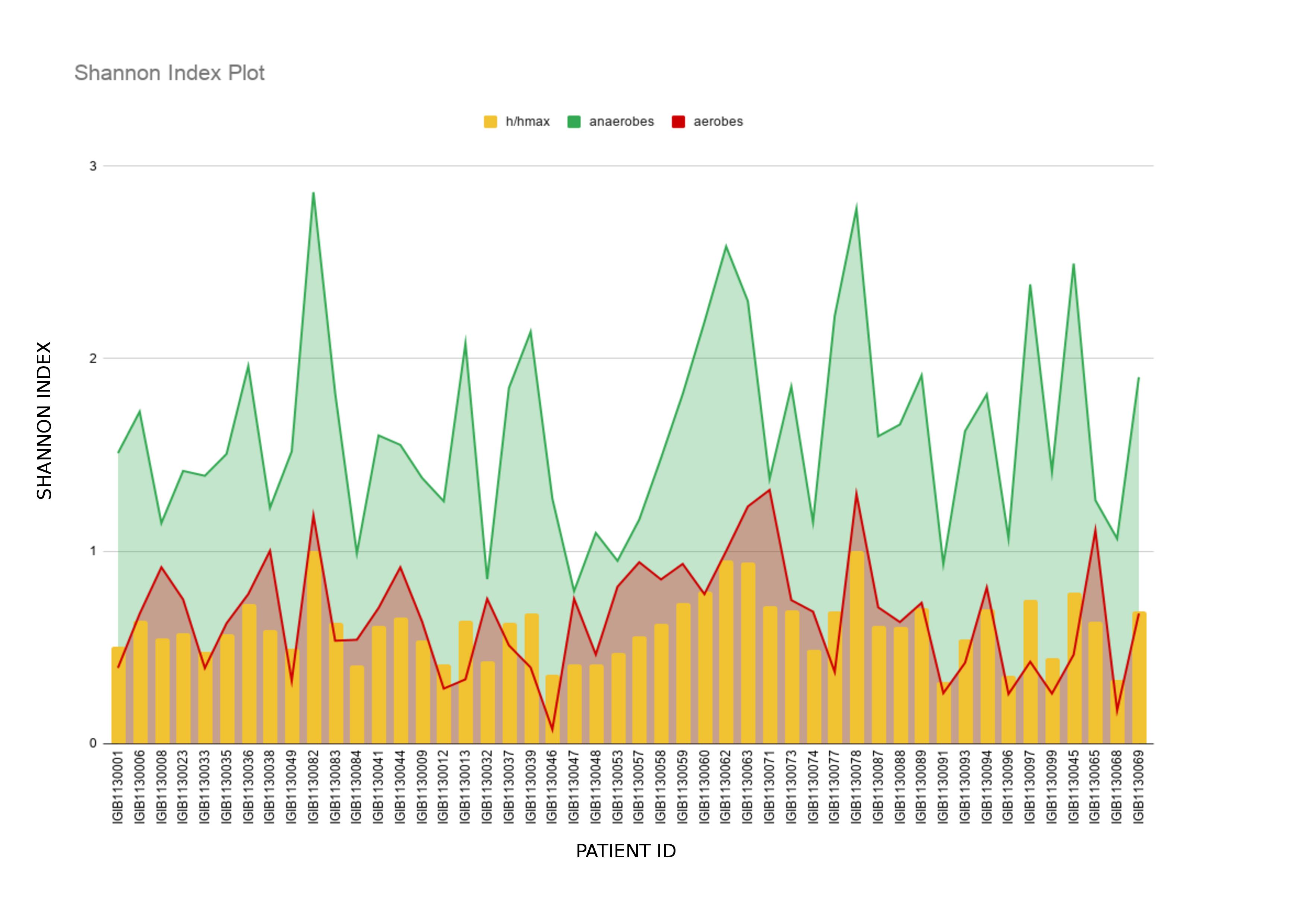

Supplement: Supplementary Figure 1 — Shannon Index plot showing bacterial abundance and evenness across patient samples. [file Data_Sheet_1.zip › SUPPLEMENTARY FIGURE 1.jpg]
